# Free triiodothyronine and global registry of acute coronary events risk score on predicting long-term major adverse cardiac events in STEMI patients undergoing primary PCI

**DOI:** 10.1186/s12944-018-0881-7

**Published:** 2018-10-12

**Authors:** Xuewei Chang, Shouyan Zhang, Mingming Zhang, Hao Wang, Caifeng Fan, Yunfei Gu, Jinghan Wei, Chunguang Qiu

**Affiliations:** 1 Department of Cardiology, the First Affiliated Hospital of Zhengzhou University, No. 1, Jianshe East Road, Zhengzhou, 450052 China; 2 Department of Cardiology, Luoyang Central Hospital Affiliated to Zhengzhou University, Luoyang, 471009 Henan China

**Keywords:** Myocardial infarction, Free triiodothyronine, Percutaneous coronary intervention

## Abstract

**Background:**

The aim of this study is to investigate the combined value of fT3 and GRACE risk score for cardiovascular prognosis in ST-segment elevation myocardial infarction (STEMI) undergoing primary percutaneous coronary intervention (PCI).

**Methods:**

Three hundred and thirty eight patients with STEMI who received successful primary PCI were enrolled in our study. All patients underwent (33.5 ± 7.1) month’s follow-up. Mace was defined as cardiac death and nonfatal myocardial infarction.

**Results:**

Multivariate Cox analysis showed that both fT3 (HR = 0.462, 95%CI: 0.364–0.587, *P* < 0.001) and GRACE score (HR = 1.011, 95%CI: 1.004–1.018, *P* = 0.003) were independent predictors of Mace. Similarly, fT3 (HR = 0.495, 95%CI: 0.355–0.690, *P* < 0.001) and GRACE score (HR = 1.022, 95%CI: 1.011–1.034, *P* < 0.001) were the most important independent predictors of cardiac death. Kaplan-Meier analysis revealed that those patients with low fT3 and higher GRACE score had higher rates of Mace (Log-Rank χ2 = 25.087, *P* < 0.001). In ROC analysis, combining fT3 and GRACE risk score had a good area under the curve (AUC) value for Mace (AUC = 0.735, 95% CI: 0.680–0.790, *P* < 0.001), with net reclassification index of 11.1 and 5.3%, respectively.

**Conclusion:**

The low fT3 level, a common phenomenon, is a strong predictor of long-term poor prognosis in STEMI patients who underwent primary PCI. The combination of GRACE score and fT3 may be a more valuable predictor of Mace as compared to each measure alone.

## Background

The prognosis of acute coronary heart disease is affected by many factors [1]. Among all neuroendocrine systems, thyroid hormone plays a major homeostatic role in modulating heart rate, cardiac contractility, and arterial peripheral resistance [2, 3]. Accumulating evidences show that abnormal thyroid homeostasis is generally associated with increased cardiovascular morbidity and mortality in patients with coronary artery disease (CAD), heart failure, stroke and so on [4–9]. Moreover, clinical and experimental evidences reveal that low free triiodothyronine (fT3) is a strong and independent predictor in patients with acute myocardial infarction [10–12].

Global Registry of Acute Coronary Events (GRACE) score was designed to predict the 6-month follow-up morbidity and mortality in patients with acute coronary syndrome [13]. Later, the ability of GRACE score in risk stratification was demonstrated to have a good predictive value up to 5 years’ follow-up [14]. Detection of a higher GRACE score was correlated with a poorer prognosis. Currently, the AHA and ESC diagnosis and treatment guidelines recommend its usage for risk evaluation in patients with acute coronary syndrome [1, 15].

Although clinical data documented that low fT3 levels and high GRACE score was closely related to major adverse cardiac events in acute myocardial infarction patients [10, 11, 16, 17], no previous research focuses on either the combined value of GRACE risk score and fT3 in ST-segment elevation myocardial infarction (STEMI) patients, or its correlation with long term follow-up Mace. Thus, the present study, for the first time, investigated whether the risk prediction and prognostic accuracy would be improved when combining the GRACE risk score and the level of fT3 in STEMI patients who underwent primary percutaneous coronary intervention (PCI). The primary endpoints involved the incidences of nonfatal myocardial infarction and cardiac death in a 3-years follow-up.

## Methods

### Study population

**Fig. 1:**
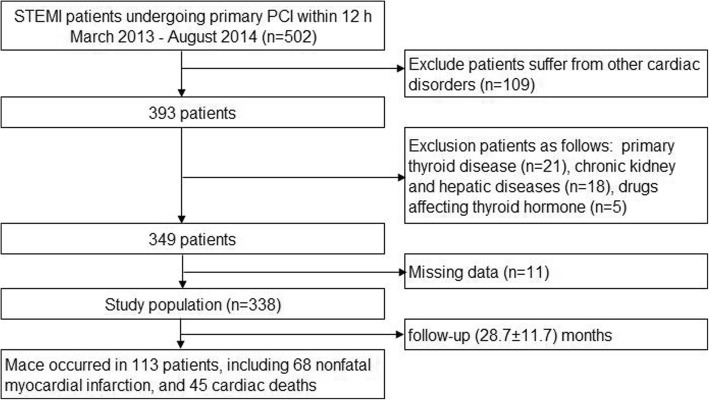
A flow chart of the study

During March 2013 to August 2014, a total of 502 consecutive STEMI patients who underwent primary PCI within less than 12 h after the onset of symptoms were enrolled in the present study. The diagnosis was based on the guidelines of the ACC/AHA for the management of STEMI, which include: typical chest pain of ischemia, persistent electrocardiographic ST elevation or new onset of complete left bundle branch block, and increasing of biomarkers of myocardial necrosis. Exclude patients with other cardiac disorders (*n* = 109) such as previous myocardial infarction (*n* = 37), history of revascularization (*n* = 29), valvular heart disease (*n* = 6), heart failure (*n* = 26) and atrial fibrillation (*n* = 11). Other exclusion criteria were as follows: primary thyroid disease (*n* = 21), chronic kidney or hepatic diseases (*n* = 18), and use of non-steroidal anti-inflammatory agents or other medicine affecting thyroid hormone levels such as amiodarone (*n* = 5). During the follow-up period, 11 patients were lost. Therefore, the final population consisted of 338 patients in this study (Fig. 1). The present study was approved by the Ethics Committee of Luoyang Central Hospital affiliated to Zhengzhou University.

### The baseline data collection

The baseline data was collected by well-trained physicians in all cases, including gender, age, body mass index, current smoker, family history, hypertension, diabetes mellitus, angina history, anterior infarction, and medication at discharge. Body mass index was calculated by dividing weight by height squared. Current smoker defined as current or former smoker of more than 1 year of duration. Hypertension was diagnosed in patients with systolic or diastolic blood pressure ≥ 140/90 mmHg or in patients using anti-hypertensive drugs. Diabetes mellitus was diagnosed as the fasting serum glucose levels above 7.0 mmol/L or under active treatment with oral hypoglycemic agents or insulin. Hyperlipidemia was defined as the patients with fasting serum total cholesterol above 5.2 mmol/L or triglyceride above 1.7 mmol/L. Angina was diagnosed to be under at least one of the following conditions: (1) Typical symptoms of angina; (2) Ischemic ST dynamic change in ECG during angina attack; (3) Confirmed by coronary angiography or CT angiography.

### Laboratory measurements

On admission, blood sampling was performed before the coronary angiography in order to measure the levels of potassium, creatinine and uric acid. Hypersensitive C reactive protein (hsCRP) was obtained from fasting blood of the next morning after admission. In addition, the levels of total cholesterol, triglyceride, and high-density lipoprotein-cholesterol were measured directly by enzymatic methods. Low-density lipoprotein cholesterol (LDL-C) was measured by Friedewald’s method. Blood was routinely measured by Beckman AU680 automated chemistry analyzer in the central laboratory of hospital. Serum fT3 level was measured by electrochemiluminescent immunoassay method using the Cobas e601 analyzer. Philips IE33 color Doppler ultrasound was employed to measure left ventricular end-diastolic dimension (LVEDD) and left ventricular ejection fraction (LVEF). Left ventricular wall thickness and LVEF were imaged under parasternal short axis view and parasternal long axis view, respectively, which was determined with Simpson’s rule. LVEDD were measured from M-mode tracings.

### Coronary angiography

All patients received dual oral antiplatelet treatment, consisting of clopidogrel (600 mg) and aspirin (300 mg) prior to primary PCI. Coronary angiography was performed using the Judkins technique by two experienced interventional cardiologists who were blinded to the study plan. Significant coronary artery disease was diagnosed visually if luminal diameter narrowing over 50% was presented in a major coronary artery. Left main coronary artery narrowing exceeding 50% was considered to be 2-vessel disease. The Gensini scoring system was utilized to describe the severity of coronary artery stenosis according to the method described previously [18]. According to the degree of stenosis, reductions of 0–25, 25–50, 50–75, 75–90, 90–99, and 100% complete occlusion were scored as 1, 2, 4, 8, 16, and 32 points, correspondingly. Each stenosed segment was assigned a multiplier according to the functional significance of the coronary artery segment. i.e. 5 for a left main lesion, 2.5 for proximal segment of left anterior descending and proximal circumflex lesions, 1.5 for a middle segment of left anterior descending lesion, 1.0 for the distal segment of left anterior descending, distal circumflex lesions, right coronary artery, posterolateral, and obtuse marginal, 0.5 for any other branches. Total Gensini score was calculated.

### The Killip classification

The Killip classification was used to predict and stratify the risk of mortality in acute myocardial infarction patients based on physical examination [19]. Patients were ranked by Killip class according to the following way: Killip class I, no clinical sign of heart failure; Killip class II, rales or crackles in lungs, an S3 and elevated jugular venous pressure; Killip class III, acute pulmonary edema; Killip class IV, systolic blood pressure lower than 90 mmHg and cardiogenic shock.

### The GRACE risk scoring system

The GRACE risk scoring system has been previously described [13]. The score was generated from variables, including age, history of myocardial infarction, systolic blood pressure, in-hospital PCI, heart rate, creatinine, ST-segment depression, elevated cardiac enzyme, and congestive heart failure (http://www.outcomes-umassmed.org). GRACE score was designed to predict the 6-month follow-up morbidity and mortality in patients with acute coronary syndrome [13]. Later, the ability of GRACE scores in risk stratification was demonstrated to have good predictive value up to five years’ follow-up [14]. Detection of a higher GRACE score was correlated with poorer prognosis. Current guidelines recommend its use for risk evaluation in patients with acute coronary syndrome [1, 15].

### Clinical outcomes

Maces were defined as nonfatal myocardial infarction and cardiac death. Nonfatal myocardial infarction was defined as the presence of typical chest pain in association with ST segment deviation, and subsequent increasing of biomarkers of serum cardiac enzyme. The definition of cardiac death required the documentation of significant arrhythmia or cardiac arrest or death attributable to congestive heart failure or myocardial infarction in absence of any other precipitating factors. Sudden unexpected death was classified as cardiac death. Deaths caused by accident were excluded. All patients were routinely followed up since first admission. Follow-up data was obtained from at least one of the following sources: face-to-face interview, hospital records, or interviewed patients or their close relatives by telephone. During (28.7 ± 11.7) months follow-up period, 11 patients were lost. Data from the 338 remaining patients were analyzed in this study. Maces occurred in 113 (33.4%) patients, including 68 (20.1%) cases of nonfatal myocardial infarction, and 45 (13.3%) cases of cardiac death. Patients were further divided into four groups according to the median value of fT3 and GRACE score: low fT3 (<3.81 pmol/L) and high GRACE score (>103) group (*n* = 103), low fT3 (<3.81 pmol/L) and low GRACE score (<103) group (*n* = 67), high fT3 (>3.81 pmol/L) and high GRACE score (>103) group (*n* = 68), as well as high fT3 (>3.81 pmol/L) and low GRACE score (<103) group (*n* = 100).

### Statistical analysis

The statistical analyses were performed using Software IBM SPSS statistics for windows version 23 (IBM Corp., Armonk, NY). All probability values were 2-tailed, and a *p* value of < 0.05 was considered statistically significant. Normal distribution of numeric data was assessed by Kolmogorov-Smirnov test. Continuous variables were expressed as mean ± standard deviation (SD). Categorical variables were presented as number and percentage. Comparisons between continuous variables were made using independent samples t-test or Mann–Whitney U test, as appropriate. Comparisons between categorical variables were evaluated using chi-square test. The relationships between fT3 and other clinical variables were assessed by Pearson test or Spearman test. Cox regression analyses were used to determine the relative risks (hazard ratio [HR]) for Mace and cardiac death associated with baseline clinical characteristics and demographic variables. To determine independent predictors of Mace and cardiac death, variables with *P* value < 0.20 in univariate analysis were entered into the multivariate Cox regression analysis. To further assess the prognostic value, Kaplan–Meier survival curve was performed to analyze the difference of Mace among groups. Then, we also generated receiver operating characteristic (ROC) curves to identify the cut-off value of fT3 and GRACE score for predicting Mace during follow-up.

## Results

### Demographic variables and baseline clinical characteristics

During the follow-up period of (28.7 ± 11.7) months, a total of 113 Maces were recorded. There was no significant difference in clinical demographic variables between Mace group and non-Mace group. However, Mace occurred most frequently in patients who had a higher Killip classification, higher GRACE score level, lower angina history, and lower serum concentration of fT3 as compared to non-Mace patients (Table 1).

**Table 1:** Demographic variables and baseline clinical characteristics

Characteristics	Mace group n=113	Non-Mace group n=225	*P* value
Age, years	61.9±11.1	59.9±12.0	0.138
Gender, male,%	74(65.5)	157(69.8)	0.458
BMI, Kg/m^2^	24.8±1.66	24.7±1.47	0.685
Current smoker ^a^	42(37.2)	69(40.7)	0.269
Hypertension	66(58.4)	114(50.7)	0.204
Diabetes mellitus	23(20.4)	52(23.1)	0.677
Medication at discharge			
Aspirin	113(100)	225(100)	1.000
Clopidogrel	113(100)	225(100)	1.000
Statins	110(97.3)	221(98.2)	0.285
Beta-blocker	102(90.2)	199(88.4)	0.713
ACEI/ARB	74(65.5)	144(64.0)	0.811
Diuretic	33(29.2)	75(33.3)	0.461
Angina history	29(25.7)	84(37.3)	0.038*
Anterior infarction	66(58.4)	112(49.8)	0.166
Killip class≥2	21(18.6)	23(10.2)	0.039*
Number of diseased vessels			0.299
1-vessel disease	69(61.1)	134(59.6)	
2-vessel disease	30(26.5)	73(32.4)	
3-vessel disease	14(12.4)	18(8.0)	
Gensini score	44.7±27.5	39.3±27.4	0.087
LVEDD, mm	48.4±4.9	48.0±4.5	0.464
LVEF, %	62.3±9.8	64.3±9.0	0.055
Creatinine, umol/L	73.9±25.8	72.3±21.9	0.567
Uric acid, mmol/L	309.7±94.4	310.9±96.8	0.916
Total cholesterol, mmol/L	4.39±1.07	4.24±0.98	0.224
Triglyceride, mmol/L	1.65±0.84	1.69±0.93	0.669
HDL-C, mmol/L	1.11±0.25	1.09±0.27	0.483
LDL-C, mmol/L	2.47±0.81	2.42±0.76	0.571
Potassium, mmol/L	4.18±0.58	4.18±0.42	0.983
hsCRP, mg/L	8.30±5.74	7.34±6.01	0.161
fT3, pmol/L	3.39±0.79	4.08±0.85	<0.001**
GRACE score	115.7±27.5	100.7±25.9	<0.001**

Values are presented as mean ± SD or n (%)

*BMI* body mass index, *ACEI* Angiotensin-converting enzyme inhibitor, *ARB* Angiotensin receptor blocker, *LVEDD* Left ventricular end-diastolic dimension, *LVEF* Left ventricular ejection fraction, *HDL-C* High-density lipoprotein-cholesterol, *LDL-C* Low-density lipoprotein-cholesterol, *hsCRP* Hypersensitive C reactive protein, *fT3* free Triiodothyronine, *GRACE score* The Global Registry of Acute Coronary Event risk score

^a^smoker defined as current or former smoker of more than 1 year of duration

*P<0.05, **P<0.001

### Associations between demographic variables and fT3

**Fig. 2:**
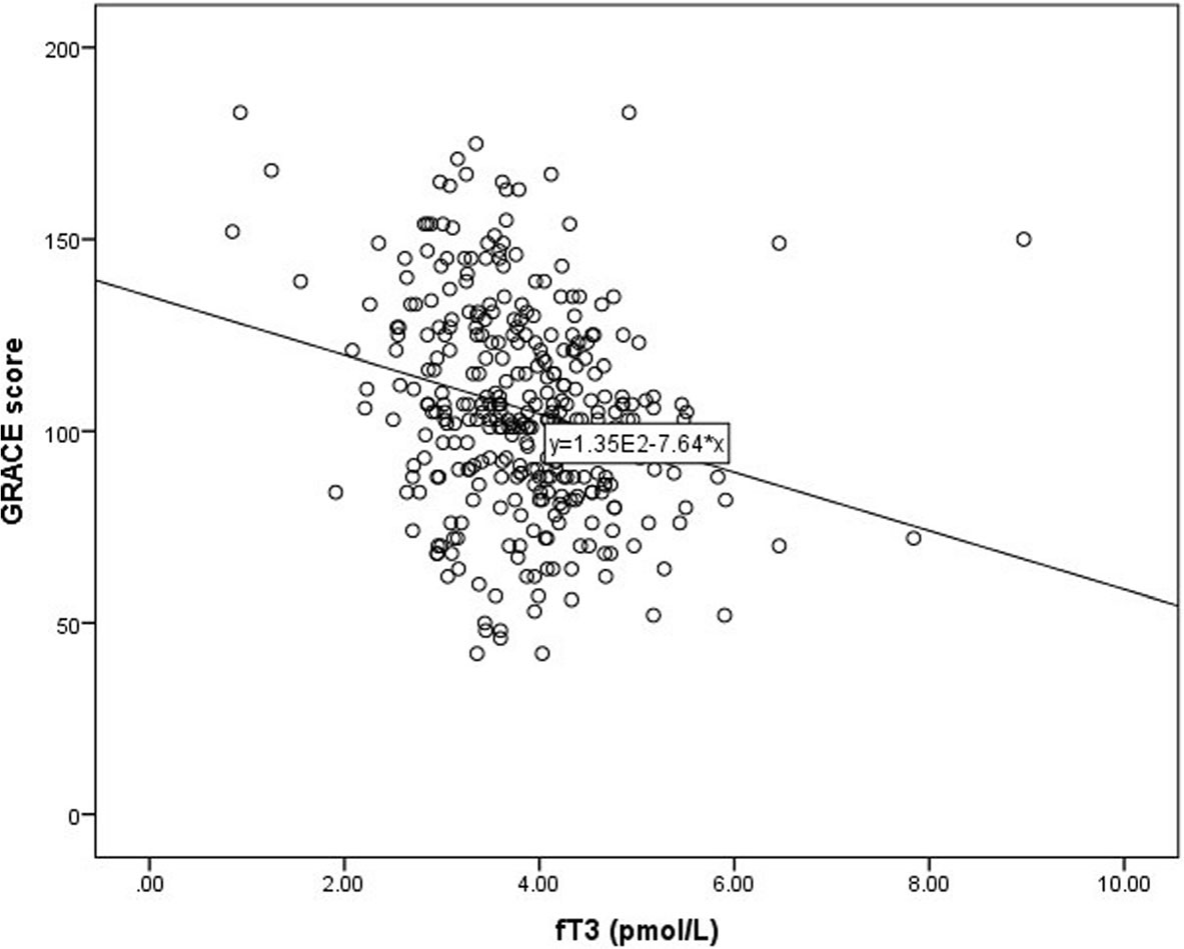
A scatter plot showing the relationship between fT3 and GRACE score

No significant association has been found between either LVEF (*r* = 0.074, *P* = 0.175), Gensini score (*r* = − 0.030, *P* = 0.586), or hsCRP (*r* = 0.017, *P* = 0.759) and fT3. However, a remarkable correlation was observed between GRACE score and fT3 (*r* = − 0.249, *P* < 0.001) (Fig. 2).

### Cox regression analysis for Mace and cardiac death

Univariate analysis of data showed that fT3, GRACE score, hsCRP, current smoker, anterior infarction, Killip class, previous angina and LVEF were strongly associated with Mace. Moreover, fT3, GRACE score, Killip class, LDL-C, hypertension and age were significantly associated with cardiac death (Table 2). A multivariate Cox regression model showed that fT3 (HR = 0.453, 95% CI 0.355–0.578, *P* < 0.001), GRACE score (HR = 1.014, 95% CI 1.006–1.021, P < 0.001), current smoker (HR = 1.782, 95% CI 1.201–2.643, *P* = 0.004), anterior infarction (HR = 1.673, 95% CI 1.163–2.407, *P* = 0.006), previous angina (HR = 0.563, 95% CI 0.365–0.869, *P* = 0.009), Killip class (HR = 1.680, 95% CI 1.032–2.737, *P* = 0.037) and were associated with Mace. Among these parameters, fT3 (HR = 0.435, 95% CI 0.297–0.638, *P* = 0.002), GRACE score (HR = 1.023, 95% CI 1.011–1.035, *P* < 0.001), LDL-C (HR = 1.587, 95% CI 1.136–2.216, *P* = 0.007), hypertension (HR = 2.315, 95% CI 1.170–4.581, *P* = 0.016), and Killip class (HR = 2.245, 95% CI 1.013–4.976, *P* = 0.046) were independent predictors of cardiac death (Table 2). After adjusting for age, current smoker, anterior infarction, Killip class and LDL-C, combination of fT3 (HR = 0.462, 95% CI 0.364–0.587, *P* < 0.001) / (HR = 0.495, 95% CI 0.355–0.690, P < 0.001) and GRACE (HR = 1.011, 95% CI 1.004–1.018, *P* = 0.003) / (HR = 1.022, 95% CI 1.011–1.034, P < 0.001) score were still significantly associated with Mace and cardiac death.

**Table 2:** Cox regression analysis for Mace and cardiac death

Variable	Univariate HR (95% CI)	P-value	Multivariatea HR (95% CI)	P-value
Mace				
fT3	0.413 (0.328-0.521)	<0.001**	0.453 (0.355-0.578)	<0.001**
GRACE score	1.017 (1.010-1.024)	<0.001**	1.014 (1.006-1.021)	<0.001**
hsCRP	1.021 (0.992-1.051)	0.158	-	-
Current smoker	1.322 (0.903-1.936)	0.152	1.782 (1.201-2.643)	0.004*
Anterior infarction	1.333 (0.917-1.939)	0.132	-	-
Killip class	1.734 (1.079-2.786)	0.023*	1.680 (1.032-2.737)	0.037*
Previous Angina	0.646 (0.423-0.985)	0.042*	0.563 (0.365-0.869)	0.009*
LVEF	0.981 (0.962-1.000)	0.047*	-	-
Cardiac death				
fT3	0.378 (0.273-0.524)	<0.001**	0.435(0.297-0.638)	0.002*
GRACE score	1.029 (1.019-1.040)	<0.001**	1.023 (1.011-1.035)	<0.001**
Killip class	1.502 (0.699-3.224)	0.197	2.245 (1.013-4.976)	0.046*
LDL-C	1.380(0.960-1.983)	0.082	1.587 (1.136-2.216)	0.007*
Hypertension	2.508(1.295-4.855)	0.006*	2.315 (1.170-4.581)	0.016*
Age	1.057(1.028-1.087)	<0.001**	-	-

*HR* hazard ratio, *CI* confidence interval, *fT3* free Triiodothyronine, *GRACE score* The Global Registry of Acute Coronary Event risk score, *hsCRP* Hypersensitive C reactive protein, *LDL-C* Low-density lipoprotein-cholesterol, *LVEF* Left ventricular ejection fraction

*P<0.05, **P<0.001

### The Kaplan–Meier survival analysis for Mace

**Fig. 3:**
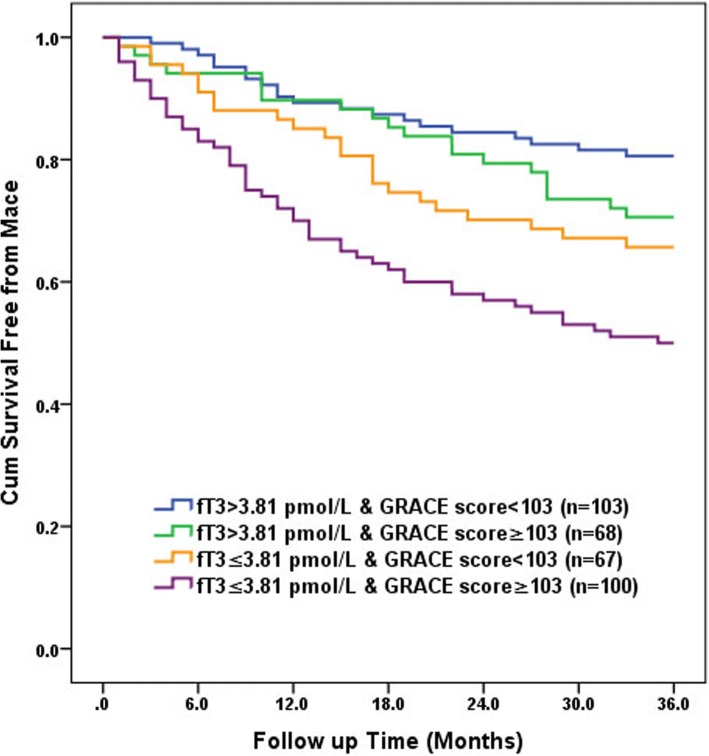
Kaplan-Meier survival analysis for Mace. All patients were stratified into 4 groups based on median values of fT3 (3.81 pmol/L) and GRACE score (103). The group with low fT3 (<3.81 pmol/L) and high GRACE score (>103) had the worse prognosis than other groups (Log-Rank χ^2^ = 25.087, *P* < 0.001). fT3: free Triiodothyronine, GRACE score: The Global Registry of Acute Coronary Event risk score, Mace: major adverse cardiac events

Patients were further divided into four groups according to the median of fT3 (3.81 pmol/L) and GRACE score (103). Results showed that patients with low fT3 and high GRACE score had the worst outcomes. Correspondingly, Kaplan–Meier survival analysis revealed that the cumulative survival rate free from Mace was the highest in the low fT3 and high GRACE score group (Log-Rank χ^2^ = 25.087, *P* < 0.001) (Fig. 3).

### Diagnostic value of fT3 and GRACE score for Mace

**Fig. 4:**
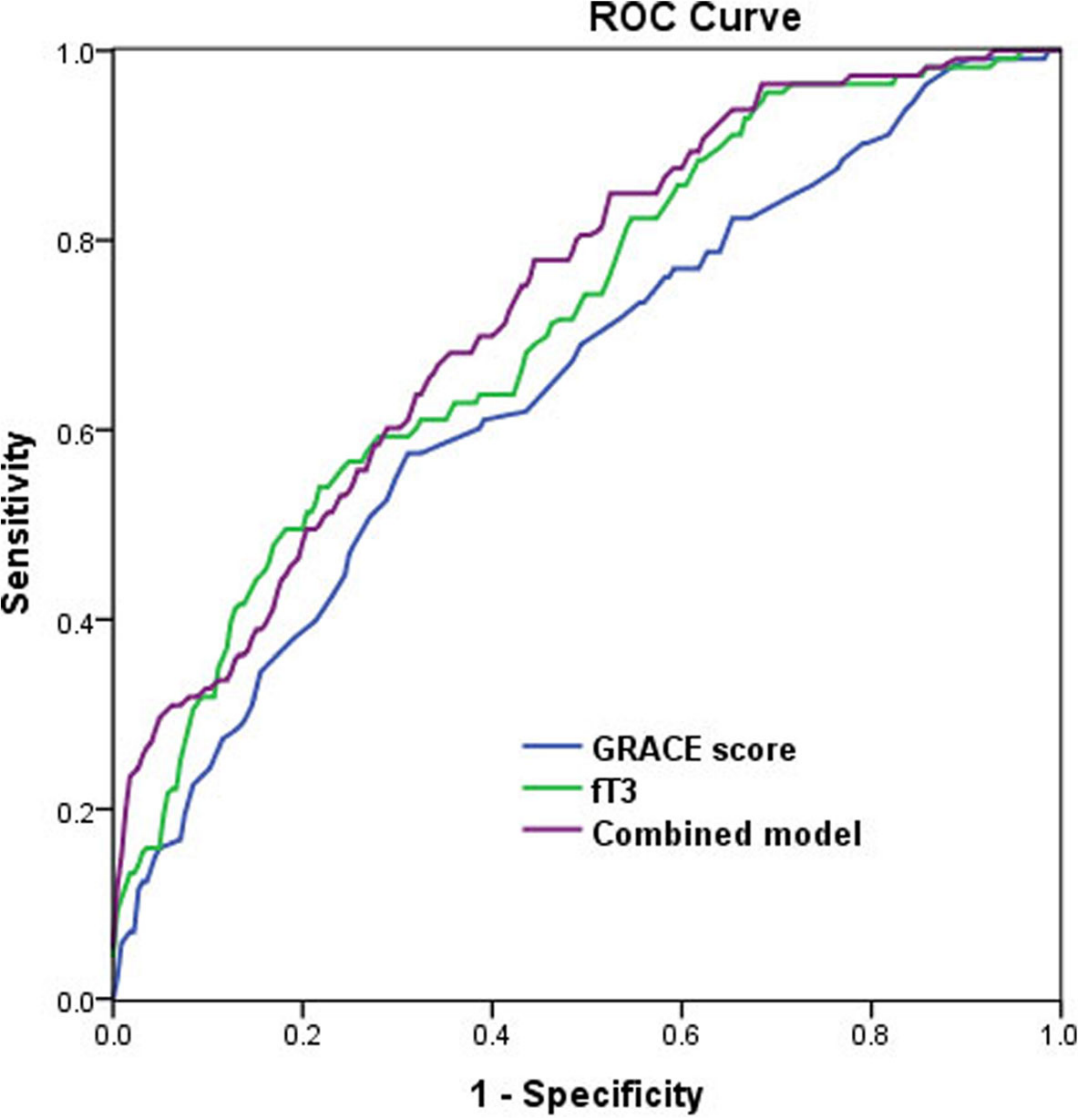
The receiver-operating characteristic (ROC) curve for free Triiodothyronine (fT3), The Global Registry of Acute Coronary Event risk score (GRACE score), and the combined value for predicting major adverse cardiac events (Mace)

ROC analysis was conducted to determine the cut-off value of fT3 and GRACE score for the prediction of Mace. The cutoff of fT3 was 3.45 pmol/L, with 54.0% sensitivity and 78.2% specificity (AUC = 0.714, 95% CI 0.657–0.771, *P* < 0.001), and the cutoff of GRACE score was 108.5, with 57.5% sensitivity and 68.9% specificity (AUC = 0.651, 95% CI 0.589–0.713, *P* < 0.001). Combining fT3 and GRACE risk score yielded to a much more valuable predictive value (AUC = 0.735, 95% CI: 0.680–0.790, *P* < 0.001) (Fig. 4). The combining model of fT3 and GRACE risk score was the one with the best correct reclassification ability, with net reclassification index of 11.1 and 5.3%, respectively).

## Discussion

Risk stratification of acute coronary syndrome is an essential part of disease management. GRACE score was the most widely used and validated risk scoring system recommend by current guidelines [1, 15]. GRACE score has been demonstrated a better discrimination in the prediction of Mace during one- and 5 years’ follow-up, including cardiac death and re-infarction [13, 14]. Our research showed that GRACE risk score can independently predict Mace in STEMI patients who underwent PCI. However, the GRACE risk score system might be limited as some certain physiological and pathological processes, such as inflammation and oxidative stress, were not wholly captured. Several previous epidemiology studies have indicated that GRACE risk score system still needs to be improved [20–22]. Klingenberg et al. reported that combinations of GRACE score and N-terminal pro-B type natriuretic peptide (NT-proBNP) could enhance risk stratification in acute coronary syndrome patients during a 12-month follow-up [21]. In another research, the author studied the effects of dickkopf-1 on 2-year follow-up prognosis in 291 acute coronary syndrome (ACS) patients, and finally found that the long-term predictive ability of GRACE score may be enhanced by adding dickkopf-1 [22]. The current study demonstrated a significant negative correlation between fT3 level and GRACE score in STEMI patients undergoing primary PCI. We have also demonstrated that the combination of fT3 and GRACE risk score was a better predictor of cardiac death and nonfatal myocardial infarction during (28.7 ± 11.7) month’s follow-up than the GRACE risk score alone.

The influence of thyroid hormones on the cardiovascular system involves the regulation of key processes related to maintenance of cardiac contractility, electrophysiological functions and cardiac structure [23]. Several studies have shown that the low fT3 patients may have an adverse prognostic impact on various acute and chronic cardiac disorders. Wang et al. studied serum levels of cardiac biomarkers and fT3 in 582 STEMI patients between January 2010 and December 2011, concluding that lower fT3 has been correlated with increased troponin T and NT-proBNP, as well as with lower LVEF [24]. Zhou et al. also reported that a strong association of fT3 levels within the normal range with Gensini score among CAD patients, concluding that fT3 levels could independently predicted the presence and severity of CAD [25]. The report by Gyu Kang et al. showed that levels of fT3 were decreased and the fT3 levels at admission were correlated with 1-year heart failure in PCI-treated acute myocardial infarction (AMI) patients [26]. Similar to the previous studies, the present study has also revealed a relationship between low serum fT3 levels and the poor prognostic of STEMI patients who underwent PCI.

Risk stratification is an essential part of disease management, and GRACE risk score represents the most widely used and validated risk scoring schemes for patients with ACS. Emerging evidence supports that the fT3 in STEMI patients as an independent risk factor for future cardiovascular events. In a previous study by Zhang et al. enrolled a total of 501 acute myocardial infarctions patients, revealed that low fT3 level is a strong predictor of short-term and long-term poor prognoses in patients with acute myocardial infarctions [11]. A recent study has shown that lower fT3 level is associated with higher mortality at 1 month and 1 year in non ST segment elevation-ACS patients [10]. Our findings were obtained from STEMI patients who underwent primary PCI demonstrating improved risk stratification using fT3 combined with GRACE score. Two recent studies found an improved risk discrimination and reclassification of patients with STEMI on addition of NT-proBNP and dickkopf-1 to the GRACE risk score system respectively [21, 22]. In our study, fT3 provides incremental information beyond the GRACE score in risk stratification for cardiac death and the composite of cardiac death and nonfatal myocardial infarction, during (28.7 ± 11.7) month’s long-term follow-up, respectively. Moreover, this is the first study demonstrates a benefit in risk stratification of STEMI patients beyond the GRACE score on addition of fT3 combined. Furthermore, we show that adding fT3 to the GRACE score improved the prognostic accuracy of the GRACE score for Mace in STEMI patients who underwent primary PCI. Additionally, the Kaplan-Meier survival curves revealed that the prognoses were poorest in the low fT3 and high GRACE risk score group than other groups.

No previous study has focused on the appropriate cut-off value of fT3 for predicting Mace in STEMI population. In this study, we estimated the association between fT3 and Mace during (28.7 ± 11.7) months follow-up in STEMI patients who underwent PCI, compared the predictive effectiveness of the combination model of fT3 and Grace score with each alone, and calculated the optimal cut-off value of fT3 in a Chinese population. In the current study, we used ROC analysis to calculate the cutoff points of fT3 based on equally weighted sensitivity and specificity. The optimal cut-off value of fT3 calculated for predicting Mace was 3.45 pmol/L. Patients with serum fT3<3.45 pmol/L have a poor prognosis. This is the first study to explore the optimal cut-off value of fT3 ratio in a Chinese STEMI population. However, the cut-off may not be applied to all CAD patients in the clinic, because sensitivity versus specificity might be altered by various factors such as the seriousness of disease.

FT3 is the most biologically active one among the thyroid hormones and affects almost every patient with serious illnesses, including acute and chronic cardiovascular diseases [27, 28]. The mechanisms underlying the low fT3 levels observed after AMI both in patients and in animal models are not fully understood. Current evidences indicated that decreased conversion from T4 to T3 in peripheral tissues due to inflammatory factors, hypoxia and hemodynamic instability result in low fT3 levels after AMI both in patients and in animal models [29, 30]. A low fT3 level may increase the incidence of short-term and long-term adverse cardiac events through several pathways. In patients with STEMI, inflammation and hypoxia are presented in heart and peripheral tissues, which are important mechanisms underlying the low fT3 levels [17, 31, 32]. A low fT3 state after STEMI changes the transcription of many cardiac structural and functional genes, for example, decreasing α-myosin heavy chain (α-MHC) and sarcoplasmic reticulum calcium-activated ATPase (SERCA2) mRNA and increasing β-MHC and phospholamban mRNA. These changes in the expression of cardiac genes are also characteristic of pathological cardiac remodeling after myocardial infarction and lead to decreased contractility of the myocardium, inhibited Ca^2+^ transport, worsened diastolic function, calcium overload, myocardial stunning and reperfusion injury [33]. In addition, thyroid hormones are powerful regulators of vasculature in the adult myocardium. Therefore, a low fT3 state would inhibit neovascularization in cardiac tissues after STEMI, which would accelerate cardiac pathologic remodeling and heart failure [28]. These changes in a low fT3 state would accelerate pathological cardiac remodeling and worsen the cardiac function, which would lead to short-term and long-term adverse cardiac events. The present study demonstrated that fT3 has independent predictive value for long-term Mace of patients with STEMI who underwent PCI. Furthermore, the levels of fT3 have an additional value to the prognostic properties of the GRACE score for the prediction of the combined endpoint of (28.7 ± 11.7) month’s mortality or nonfatal myocardial infarction.

Thyroid hormones have significant effects on synthesis, mobilisation and metabolism of lipids through multiple mechanisms. Interestingly, a series of studies reported that thyroid hormones were closely associated with an increase of lipid abnormalities [16, 34]. However, this issue remains controversial, as several studies failed to observe the same association [10, 11, 32]. Our study didn’t find any possible relationship between lipids and fT3, either. This might be due to that the level of fT3 changes sensitively and rapidly with the alteration of acute stress, inflammation and hypoxia existing in hearts and peripheral tissues of STEMI patients. However, the influences induced by the factors mentioned above on lipid metabolisms would present relatively later. In addition, the levels of lipid are affected by various factors. Scicchitano P et al. [35] reported that nutraceuticals and functional food ingredients should be considered to influence lipids levels in patients with CAD. Therefore, the present study did not observe the relationship between serum lipids and fT3.

It is still unclear if low fT3 is directly related to increased mortality or it is only a sign of more diffuse and active atherosclerosis which is the underlying cause of mortality. In hypothyroidism characterized by low fT3 levels, impaired lipid metabolism could explain for the increased atherosclerosis rates. But as to low fT3, the situation is different because TSH and T3 levels are most likely normal before acute onset of the disease. Therefore, it is not reasonable to consider that increased atherosclerosis is due to impaired lipid metabolism in low fT3 patients [10]. Our findings support this hypothesis. Further studies should be conducted to research in this field.

All available data indicate that alterations in thyroid function tests are not uncommon in STEMI patients. The low fT3 levels represent a hormonal imbalance that may significantly influence pathophysiological mechanisms and cardiovascular hemodynamics. This altered thyroid state, and, more specifically, the reduction of fT3, seems to be related with overall worse prognosis. The present study showed that combination of GRACE score and fT3 may be a simple and valuable independent predictor for cardiac death and Mace in patients with STEMI undergoing primary PCI, which may be a supplementary to the current risk stratification tools and may indicate long-term clinical outcomes. Our results could be useful in the prognostic stratification of patients suffering an ACS, and may have potential implications for improving the development of novel therapies.

The current study has several limitations. Firstly, patients enrolled in this study came from a single center in China, which might limit the extrapolation of our results if regard to race, and the sample size was relatively small. Secondly, the population was limited to STEMI patients underwent primary PCI, thus, the results may not apply to all STEMI patients. Additionally, this study did not measure the fT3 level during follow-up, whether replacing thyroid hormones and raising fT3 levels into the normal range could help to improve the outcomes of the patients in the low fT3 group is unknown. Therefore, multi-center studies may provide different insight. Finally, potential sources of heterogeneity in follow-up protocols might also lead to biases. Eleven patients were lost during the follow-up period in the present study. It is unavoidable in epidemiological studies that may be biased toward the null hypothesis because of the lost cases might have more extreme values for the analyzed variables.

## Conclusion

The present study revealed that combining predictive value of GRACE score and fT3 may be a more valuable predictor of Mace as compared to each measure alone. It is important, though, to emphasize that low fT3 in STEMI patients seem to have worse outcome. In absence of sufficient data through large-scale clinical studies, it remains unclear whether low fT3 is directly linked to worse prognosis or it constitutes a marker of the severity of illness. In addition, it is still controversial whether routine administration of levothyroxine is associated with lower mortality or better prognosis in patients with STEMI. Our results may have potential implications for risk stratification and may indicate long-term clinical outcomes. Nevertheless, more high-quality studies are needed to determine whether thyroid replacement therapy could reduce the mortality of low fT3 patients.

## Abbreviations

CAD: Coronary artery disease
fT3: Free triiodothyronine
GRACE: Global Registry of Acute Coronary Events
MACE: Major Adverse Cardiac Events
NT-proBNP: N-terminal pro-B type natriuretic peptide
PCI: Primary percutaneous coronary intervention
STEMI: ST-segment elevation myocardial infarction
